# Dynamin-like proteins in *Trypanosoma brucei*: A division of labour between two paralogs?

**DOI:** 10.1371/journal.pone.0177200

**Published:** 2017-05-08

**Authors:** Corinna Benz, Eva Stříbrná, Hassan Hashimi, Julius Lukeš

**Affiliations:** 1 Faculty of Sciences, University of South Bohemia, České Budějovice (Budweis), Czech Republic; 2 Institute of Parasitology, Biology Centre, Czech Academy of Sciences, České Budějovice (Budweis), Czech Republic; 3 Canadian Institute for Advanced Research, Toronto, Canada; University of Cambridge, UNITED KINGDOM

## Abstract

Dynamins and dynamin-like proteins (DLPs) belong to a family of large GTPases involved in membrane remodelling events. These include both fusion and fission processes with different dynamin proteins often having a specialised function within the same organism. *Trypanosoma brucei* is thought to have only one multifunctional DLP (*Tb*DLP). While this was initially reported to function in mitochondrial division only, an additional role in endocytosis and cytokinesis was later also proposed. Since there are two copies of *Tb*DLP present in the trypanosome genome, we investigated potential functional differences between these two paralogs by re-expressing either protein in a *Tb*DLP RNAi background. These paralogs, called *Tb*DLP1 and *Tb*DLP2, are almost identical bar a few amino acid substitutions. Our results, based on cell lines carrying tagged and RNAi-resistant versions of each protein, show that overexpression of *Tb*DLP1 alone is able to rescue the observed endocytosis and growth defects in the mammalian bloodstream form (BSF) of the parasite. While *Tb*DLP2 shows no rescue in our experiments in BSF, this might also be due to lower expression levels of the protein in this life stage. In contrast, both *Tb*DLP proteins apparently play more complementary roles in the insect procyclic form (PCF) since neither *Tb*DLP1 nor *Tb*DLP2 alone can fully restore wildtype growth and morphology in *Tb*DLP-depleted parasites.

## Introduction

*Trypanosoma brucei* is an important single-celled parasite that causes African trypanosomiasis in humans and the wasting disease nagana in cattle. The flagellate is transmitted between mammalian hosts by an insect vector, the tsetse fly. During its development and transmission cycle, *T*. *brucei* goes through a variety of morphological and metabolic changes that largely depend on its environment [1]. Whereas the mammalian-dwelling long slender bloodstream form (BSF) flagellates rely solely on glycolysis for energy generation, the main energy source of the insect procyclic form (PCF) are amino acids, such as proline [2]. This metabolic adjustment requires activation of the mitochondrion, with which PCF cells generate most cellular ATP via oxidative phosphorylation, thus making the organelle more essential for this life cycle stage [3]. Mitochondrial morphology is also dramatically altered during this inter-stagial transformation, changing from a simple tube-like appearance in BSF to an elaborate and highly branched structure with multiple cristae in PCF [4,5].

While the advantages of living in a nutrient-rich environment are obvious, the mammalian bloodstream also presents BSF parasites with a number of challenges. The highly active, adaptive immune response of the host produces specific antibodies against the prevalent variant surface glycoprotein (VSG) of the parasite, and only periodic switching to another variant from the vast VSG reservoir present in its genome allows *T*. *brucei* to survive [6,7]. Another mechanism to escape immunological detection is the dramatic upregulation of endocytosis in BSF as compared to PCF, which facilitates surface coat recycling [8]. This rapid recycling mechanism has potentially evolved to clear VSG-bound host antibodies from the cell surface [9] and is crucial for trypanosome survival. Any disturbance of the endocytic process, such as caused by depletion of the major endocytic vesicle coat protein clathrin [10] or the cytoskeletal protein actin [11] is rapidly lethal in BSF. In trypanosomes, all exo- and endocytosis occurs through a single invagination of the plasma membrane located at the posterior end of the cell, the flagellar pocket, and hence endocytosis defects are rapidly manifested as a visible swelling of this pocket [12]. This so-called ‘big eye’ phenotype or flagellar pocket enlargement has also been observed in PCF upon depletion of clathrin [10]. However, it does not appear to be the primary or most important defect in this stage because an accumulation of intracellular vesicles and a general rounding up of the cells was also observed [10].

**Fig 1:**
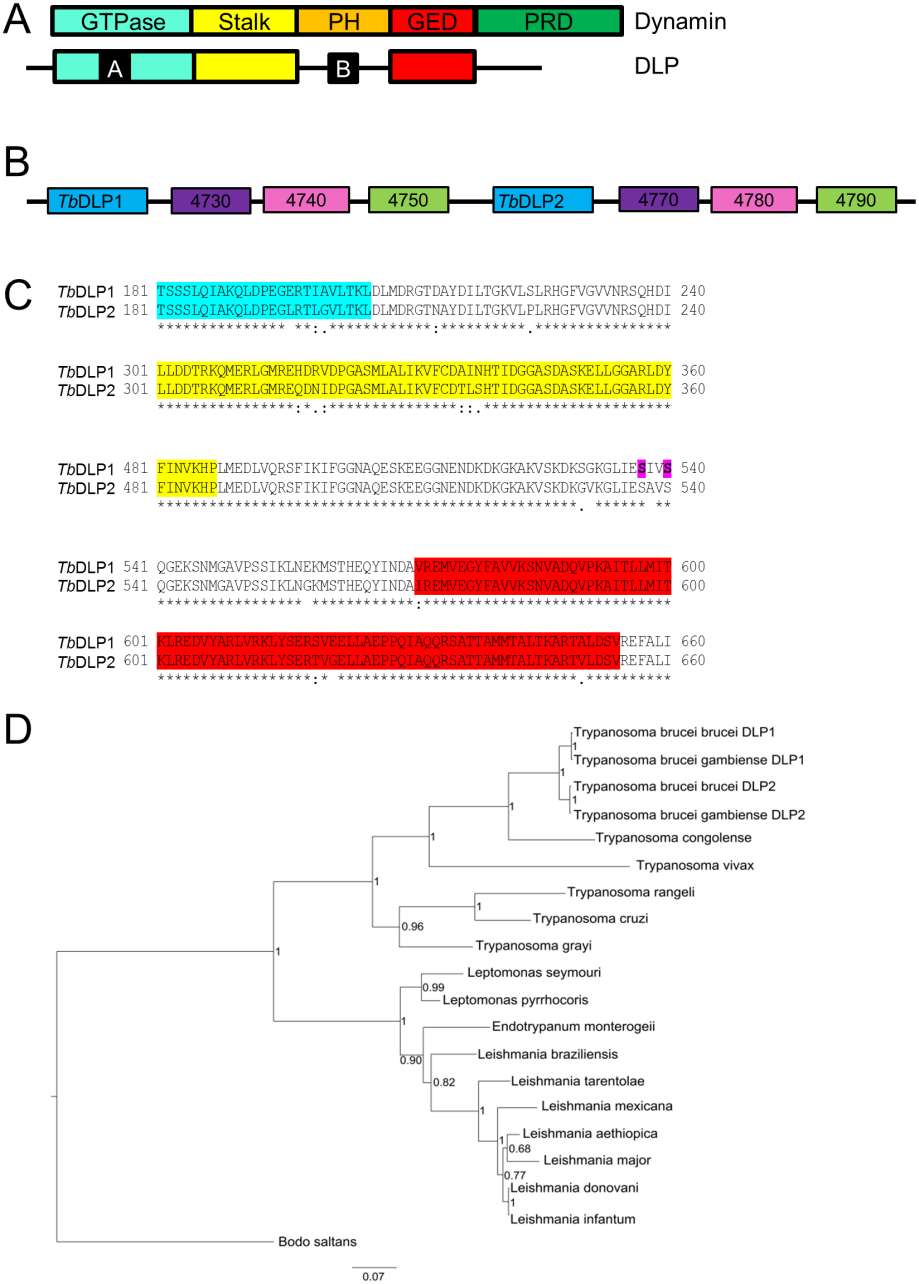
Bioinformatics analyses of *Tb*DLP1 and *Tb*DLP2. A) Domain structure of a typical dynamin and DLP. Dynamin consists of five different domains, an N-terminal GTPase domain (light blue), a middle domain or stalk (yellow), a PH domain (orange), a GTPase effector domain (GED, red) and the C-terminal proline-rich domain (PR, green). DLPs lack the PH and PR domains, but have additional inserts A and B (black). B) Scheme of the genomic locus of *Tb*DLP1 and *Tb*DLP2 on chromosome 3 (not to scale). *Tb*DLP1 and *Tb*DLP2 are in blue. Paralogous genes are indicated by the same colour and gene names were abbreviated to the final 4 numbers. Tb927.3.4730 and Tb927.3.4770: LRTP, purple boxes; Tb927.3.4740 and Tb927.3.4780: conserved hypothetical protein, pink boxes; Tb927.3.4750 and Tb927.3.4770: putative aminopeptidase M1, green boxes. C)Clustal Omega alignment of variable regions of *Tb*DLP1 and *Tb*DLP2. An alignment of *Tb*DLP1 and *Tb*DLP2, highlighting the 19 amino acid variants. Colours indicate the domain these variants are found in and are the same as in A). Serine residues highlighted in pink mark phosphorylation sites in *Tb*DLP1. D) Phylogenetic tree of trypanosomatid DLP proteins. Genus and species names identify organisms from which a single DLP ortholog was identified, while the paralog names append *T*. *brucei* subspecies names. The free-living kinetoplastid *B*. *saltans* is included as an outgroup. Numbers adjacent to nodes indicate calculated Bayesian posterior probabilities. Scale bar: substitutions per site.

Endocytosis in BSF and mitochondrial division in PCF parasites belong to the most important cellular processes for the respective life stage and both are generally regulated by a GTPase of the dynamin family. Dynamins and dynamin-like proteins (DLPs) belong to a group of well conserved large GTPases with important functions in endocytosis and in the division of organelles such as mitochondria and peroxisomes. The group also includes fusion GTPases, such as the inner mitochondrial membrane protein optic atrophy 1 (OPA1) and the outer mitochondrial membrane GTPases mitofusin 1 (Mfn1) and mitofusin 2 (Mfn2) [13]. Upon contact with membranes, dynamin oligomerizes and forms spirals, the constriction of which eventually leads to fission [14]. Structurally, dynamins and DLPs consist of an N-terminal GTPase domain, a middle stalk domain which is important for dimerization and a C-terminal GTPase effector domain (GED) (Fig 1A). In contrast to dynamins, DLPs have no transmembrane or lipid-interacting Pleckstrin homology (PH) domains and the proline-rich domain near the C-terminus is also missing (Fig 1A). Instead, DLPs have an additional insert “B”, which might be important for interaction with adaptors that specifically target them to a certain organelle [14]. Moreover, insert B is a hotspot for post-translational modifications [15] and splice variants [16]. Lacking any transmembrane or lipid-interacting domains, DLPs are soluble cytosolic proteins that have to be recruited to mitochondria and peroxisomes by certain adaptor proteins [17]. Whereas dynamins are extremely well conserved, their adaptor proteins are not, and differ significantly from organism to organism. Mitochondrial fission 1 (Fis1), mitochondrial fission factor (Mff), mitochondrial dynamics proteins of 49 kDa (Mid49) and 51kDa (Mid51) are DLP adaptors found in mammalian cells [18], while Fis1, mitochondrial division protein 1 (Mdv1) and Caf4 play the same role in yeast [19]. The role of Fis1 in mitochondrial division in mammals remains controversial [18], and it might only be needed for mitochondrial targeting of DLP in certain stress situations and/or specific tissues [20].

With a shared function in membrane remodelling, dynamins are found in all kingdoms of life including bacteria. Most prokaryotes encode at least two dynamin or DLP variants, which can be arranged in an operon [21]. If two paralogs are present, they most likely result from a gene duplication event and in some bacterial species have even fused into a single protein [21]. While the function of bacterial dynamins has not been investigated in great depth, *Bacillis subtilis* DynA was found to promote membrane fusion and was localised to the site of septation [22]. If present, both bacterial dynamin variants have to cooperate to be active, which is reminiscent of the outer mitochondrial membrane fusion dynamins Mfn1 and Mfn2 in mammals. These proteins are highly related, but functionally non-redundant [23].

Previous work on trypanosome DLP initially suggested that it was involved solely in mitochondrial division [24]. Here, it is important to note that *T*. *brucei* cells invariably carry a single mitochondrion, which therefore needs to be properly replicated and faithfully segregated during cytokinesis to generate viable progeny [25, 26], hence rendering mitochondrial fission essential for parasite survival. *Tb*DLP remains the only identified mitochondrial fission protein in *T*. *brucei* to date, and nothing is known about the mechanism and/or regulation of this highly important process. How exactly mitochondrial fission is coordinated with the rest of the cell cycle has also not been explicitly investigated. It was shown, however, that the depletion of *Tb*DLP in PCF also causes a rather dramatic disruption of endocytosis and flagellar pocket enlargement in addition to effects on mitochondrial division and cytokinesis [27]. These authors also suggested that the cytokinesis defects arise as a consequence of a specific cell cycle checkpoint rather than a mechanistic cytokinesis block caused by the undivided and hence trapped mitochondrion. So is *Tb*DLP indeed a multifunctional dynamin as suggested or is there more to the story than meets the eye?

From an evolutionary point of view, the last eukaryotic common ancestor (LECA) is thought to have had a bifunctional dynamin responsible for both endocytosis and organelle division, which further diversified during evolution [28]. According to this analysis, functional specialization of dynamins and the development of different isoforms occurred at least on three independent occasions. This may also be the case—at least to some extent—in the highly diverged excavate *T*. *brucei* and related trypanosomatid protists. Reminiscent of the situation in bacteria, the trypanosome DLP locus appears to have been duplicated and, given the difference in amino acid sequence between the *Tb*DLP1 and *Tb*DLP2 paralogs, we wondered whether they already functionally diversified.

Proteomic evidence for the specific expression of *Tb*DLP1 and *Tb*DLP2 is readily available in the literature (summarized in Table 1) and suggests a potentially differential expression of *Tb*DLP1 and *Tb*DLP2 between BSF and PCF stages, as well as specific post-translational modifications [29–34]. To more easily compare the different studies, we arbitrarily set the expression of either *Tb*DLP to 100% in the proliferative long slender BSF and calculated the other values accordingly. In summary, *Tb*DLP1 was consistently less abundant in the PCF (with values varying between 22 and 74% in different studies; Table 1). Interestingly, this isoform appears to be slightly upregulated in the other short stumpy BSF stage, a quiescent form that is competent for uptake by the tsetse fly to complete the parasite’s life cycle [1].

**Table 1:** Summary of available *Tb*DLP proteomics data.

	*Tb*DLP1 (Tb927.3.4720)	*Tb*DLP2 (Tb927.3.4760)	Reference
Abundance	LS:100% SS:153% PCF:40%	LS:100% SS:457% PCF:310%	[31]
	LS:100% SS:140% PCF:74%	LS: 100% SS: 560% PCF: 500%	[30]
	BSF: 100% PCF: 40%	Not regulated*	[29]
	BSF:100% PCF: 30%	BSF: 100% PCF: 126%*	[33]
	BSF: 100% PCF: 22%	BSF: 100% PCF: 116%*	[34]
Post-translational modifications	S537-P S540-P (BSF)	-	[32] [33]

Proteomics data as available through www.tritrypdb.org was analysed according to the specificity of the observed peptides for *Tb*DLP1 or *Tb*DLP2. Only specific peptides were considered for the abundance changes and the values for the long slender (LS) BSF stage arbitrarily set to 100%. SS indicates short stumpy BSF;

* denotes PCF grown in SDM79 medium containing glucose.

Both phosphorylation sites on *Tb*DLP1 were identified in both studies mentioned.

In contrast, *Tb*DLP2 levels increased around 5-fold during differentiation from long slender to short stumpy BSF and remained high in PCF in two studies [30, 31], while three other studies reported that the level of *Tb*DLP2 remained either unaltered [30] or upregulated to a minor extent [33, 34] (Table 1). However, it should be noted that in the reports in which no *Tb*DLP2 upregulation was observed in PCF, these cells were grown in SDM79 media containing 6 mM glucose [35], which is a preferred energy source over the amino acids that are normally consumed in the wild [36]. The proteomic studies demonstrating pronounced *Tb*DLP2 upregulation in PCF utilized media in which glucose was absent, thus requiring an active mitochondrion for the catabolism of proline [36], and potentially also components of its fission machinery.

Minor differences in amino acid sequence could potentially change the specificity of *Tb*DLP interactions with corresponding adaptor proteins or the availability for post-translational modifications and hence affect stability of either paralog in a given life stage. Alternatively, targeting to either the flagellar pocket or the mitochondrion could thus be regulated. Our results show that while *Tb*DLP1 alone is essential and sufficient for BSF trypanosomes, their PCF counterparts depend on both *Tb*DLPs for normal growth, endocytosis and mitochondrial morphology.

## Materials and methods

### Cell lines, cultivation and growth curves

BSF single marker (SM) and PCF 29–13 cells [37] were cultured essentially as described elsewhere [38]. BSF and PCF clones bearing p2T7-177 derived RNAi constructs [39] and pT7V5- based overexpression constructs [40] were induced with 1 μg/ml tetracycline. For growth measurements, BSF and PCF cell lines were induced at a density of 1 x 10^5^/ml and 1 x 10^6^/ml, respectively, counted daily and diluted back to the initial density as appropriate.

### Plasmids, transfections and selection

For creating the *Tb*DLP RNAi plasmid, a fragment of the N-terminal part of the open reading frame (ORF) (nts 75–512) was amplified with primers CB36 and CB38 (S1 Table), digested with BamHI and XhoI and cloned into p2T7-177 [39] to create plasmid pCR56. It should be noted that the amplified fragment lies within the part of *Tb*DLP1 and *Tb*DLP2 that is exactly identical at the nucleotide level. To specifically add a tag to the N-terminus of either *Tb*DLP at their endogenous locus, a fragment of the N-terminal part of the ORF (nts 3–612) was amplified from *T*. *brucei* 427 genomic DNA using primers CB48 and CB29 (S1 Table), digested with BglII and HpaI and cloned into compatible BamHI-EcoRV sites of p2678 [41], thus creating plasmid pCR19. These sites were chosen because the *Tb*DLP ORF contains multiple sites for the most commonly used restriction enzymes, thus necessitating shortening of the protein C part of the PTP-tag in the vector. This modification hinders detection of the tagged protein with anti-protein C antibody as well as tandem affinity purification of the tagged *Tb*DLPs, while still allowing single-step purification using IgG-Sepharose, which binds to the protein A part of the tag. The construct was linearized with HindIII (cutting after nt 67), thus creating a tagged protein without interfering with the specific parts of either *Tb*DLP. For the *Tb*DLP rescue constructs, a recoded and hence RNAi-refractory N-terminal part of the protein (nts 1–572) was synthesised by ShineGene Molecular Biotech (China). This N-terminal part was amplified with primers CB69 and CB70 (S1 Table) from the provided plasmid (the created fragment encompassed nts 1–512), and then digested with HindIII and EcoRV. Next, it was ligated together with a C-terminal fragment of *Tb*DLP (nts 513–1983) amplified with primers CB67 and CB68 (S1 Table) and digested with EcoRV and BglII into HindIII-BamHI sites of pT7V5 [40]. The identity of the amplified *Tb*DLP was verified by sequencing of the entire constructs (pCR40 = *Tb*DLP1-V5; PCR55 = *Tb*DLP2-V5).

Both overexpression plasmids and the RNAi plasmid were digested with NotI for linearization, while pCR19 for endogenous tagging was linearized utilising an internal HindIII site. All constructs were transfected into BSF SM or PCF 29–13 cells using the Amaxa Nucleofector II electroporator and programs X-001 (BSF) and X-014 (PCF), respectively. Selection was with 5 μg/ml phleomycin (pCR56 [*Tb*DLP RNAi]), 0.5 μg/ml puromycin (pCR19 [PTP- *Tb*DLP], pCR40 [*Tb*DLP1-V5] and pCR55 [*Tb*DLP2-V5]) for PCF and 0.2 μg/ml phleomycin (pCR56 [*Tb*DLP RNAi]), 0.2 μg/ml puromycin (pCR19 [PTP- *Tb*DLP], pCR40 [*Tb*DLP1-V5] and pCR55 [*Tb*DLP2-V5]) for BSF.

### Identification of endogenously tagged *Tb*DLPs

The endogenous tagging construct (pCR19) was designed to integrate into either of the *Tb*DLP loci without changing the coding sequence of the targeted protein. To identify which protein was tagged with PTP, genomic DNA was isolated from all BSF and PCF clones expressing a tagged protein of the correct size as shown by western blot analysis, using the Qiagen DNA extraction kit and following the manufacturer’s instructions. PCR amplification was then performed using forward primer CB30 (binding within the PTP tag) and reverse primers CB31 (specific for the *Tb*DLP1 intergenic region 3’ of the ORF) and CB32 (specific for the *Tb*DLP2 intergenic region 3’ of the ORF). Clones expressing PTP-*Tb*DLP1 and PTP-*Tb*DLP2 were thus identified in both life cycle stages (S1 Fig; details of oligos in S1 Table).

### Northern and western blot analysis

Ten μg per sample of total RNA was isolated from BSF or PCF cells, resolved on a formaldehyde gel that was blotted onto nitrocellulose membrane by overnight capillary transfer. This membrane was hybridized as previously described [42], using a 5’-end, ^32^P-labelled antisense probe generated from the CB36 and CB38 amplicon template.

Trypanosome cell lysates were prepared by boiling in 1x Laemmli buffer and the equivalent of 2 x 10^6^ cells was loaded per lane onto an SDS PAGE gel. Proteins were transferred to nitrocellulose or PVDF membranes using wet transfer. Following a blocking step in 5% milk-phosphate-buffered saline (PBS) for 1 hr at room temperature, blots were incubated in primary antibody overnight at 4°C. After two 5 min washes in PBS, blots were incubated with the appropriate horse radish peroxidase (HRP)-coupled secondary antibody (α-mouse or α -rabbit; used at 1:2,000 dilution; Sigma) at room temperature for 1 hr before two further washes in PBS. Signals were developed using the Clarity ECL substrate (BioRad, USA). The following primary antibodies were used in this study: α -protein A (produced in rabbit; used at 1:20,000; Sigma-Aldrich), α -V5 (produced in mouse; used at 1:1,000; Invitrogen), α -enolase (produced in rabbit; used at 1:2,000; gift from Paul A.M. Michels), α -HA (produced in mouse, used at 1:1,000, Sigma-Aldrich). Band intensities were quantified using Image J software [43].

### Immunofluorescence

BSF and PCF parasites were incubated with 100 nM MitoTracker Red CMXRos (Molecular Probes) for 30 min at 37 and 27°C, respectively, washed and spread onto glass slides. After 10 min of drying, slides were put into methanol at -20°C for 1 hr before rehydration in PBS. Slides were then incubated in α -protein A antibody (used at 1:50,000; Sigma-Aldrich) for 1 hr at room temperature. Following two 5 min washes in PBS, the secondary antibody (goat α -rabbit AlexaFluor488; used at 1:200; Molecular Probes) was added to the slides which were incubated in the dark for 1 hr at room temperature. After two 5 min washes in PBS, DAPI (used at 1 μg/ml; Sigma-Aldrich) was added for 5 min and washed off again with two 5 min incubations in PBS. Coverslips were applied to the slides and fixed with nail polish. Slides were examined using an Axioscope 2 fluorescent microscope (Zeiss).

### Electron microscopy, DAPI staining and big eye counts

For transmission electron microscopy (TEM), pelleted BSF and PCF cells were processed as described elsewhere and observed in a JEOL 1010 TEM at an accelerating voltage of 80 kV [42].

Slides were prepared at the same times as growth was recorded and stored in -20°C methanol for further analysis. Following rehydration in PBS, a DAPI solution (final concentration 1 μg/ml) was applied and slides were immediately examined under a fluorescent microscope. At least 200 cells per slide were counted and scored according to their number of nuclei and kinetoplasts. From the same slides, at least 200 phase contrast images were scored as to whether or not they had a big eye phenotype (enlargement of the flagellar pocket area). Experiments were performed in triplicate for every cell line and time point analysed. Error bars are standard deviations from three biological replicates.

### Bioinformatics

*Tb*DLP (Tb927.3.4720 and Tb927.3.4760) sequences were retrieved from the TriTrypDB website (www.tritrypdb.org) and aligned using Custal Omega [44]. The cartoon version of the *Tb*DLP locus was generated in PowerPoint (Microsoft) and information within obtained from the TriTrypDB website (www.tritrypdb.org).

Kinetoplastid DLP homologues were identified and retrieved from the TriTrypDB website (www.tritrypdb.org) using the *T*. *brucei* sequence as a query. The *Bodo saltans* sequence, which was used as an outgroup, was obtained from the Sanger Institute database (http://www.sanger.ac.uk/resources/downloads/protozoa/bodo-saltans.html). A list containing the accession numbers of the kinetoplastid DLPs is provided in S2 Table. The full dataset was then aligned in MAFFT [45] using the localpair algorithm. The alignment was visually inspected and ambiguously aligned regions were removed in Seaview 4 [46]. The maximum likelihood phylogenetic topology was inferred using RAxML 8.28 [47] under the LG+G model. The best-scoring tree as well as non-parametric bootstrap support was estimated using the fast-bootstrap algorithm (-f a) from 1000 replicates. Bayesian posterior probabilities as another mean of branching support were computed in MrBayes 3.2 [48]. Four independent MCMC chains were run for 3x10^6^ generations under the above mentioned model and default chain parameters, then checked for convergence and after omitting the first 5x10^5^ generations as burn-in, topology and posterior probabilities were reconstructed.

## Results

### Genomic organisation

Within the genomic locus of the two trypanosome DLPs (*Tb*DLP1:Tb927.3.4720 and *Tb*DLP2:Tb927.3.4760), there are three additional genes that are also duplicated (Fig 1B). Comparing all of these ORFs at the nucleotide level as annotated in TriTrypDB (www.tritrypdb.org) shows 100% conservation for the other three ORFs (Tb927.3.4730/Tb927.3.4770: LRTP, Tb927.3.4740/Tb927.3.4780: conserved hypothetical protein, Tb927.3.4750/Tb927.3.4790: putative aminopeptidase M1) and even the intergenic regions between them are well conserved. The only differences within the entire locus are found in the coding sequences of *Tb*DLP1 and *Tb*DLP2, resulting in 19 amino acid variations in the final proteins (Fig 1C). These differences are real and were corroborated by us when sequencing the rescue and overexpression constructs produced in this study (data not shown). The three variations found within the GTPase domain are either conserved (I199L, A200G) or not found in residues important for activity when compared to human dynamin 1 (E196L) [49]. Most other variations are also conserved with the notable exception of the G531V and I538A substitutions which are found within the insert B region. *Tb*DLP1-specific phosphosites were found adjacent to these in two independent studies [32, 33]. The two serine residues concerned (S537 and S540) are also present in *Tb*DLP2 but were not found to be post-translationally modified in this protein.

Duplication of *Tb*DLP appears to have only occurred in the *T*. *brucei* subclade, as evidenced by other sequenced trypanosomatids and the free-living kinetoplastid *Bodo saltans* having only one copy of the full *DLP* ORF within their respective genomes (Fig 1D).

### Evidence of expression

**Fig 2:**
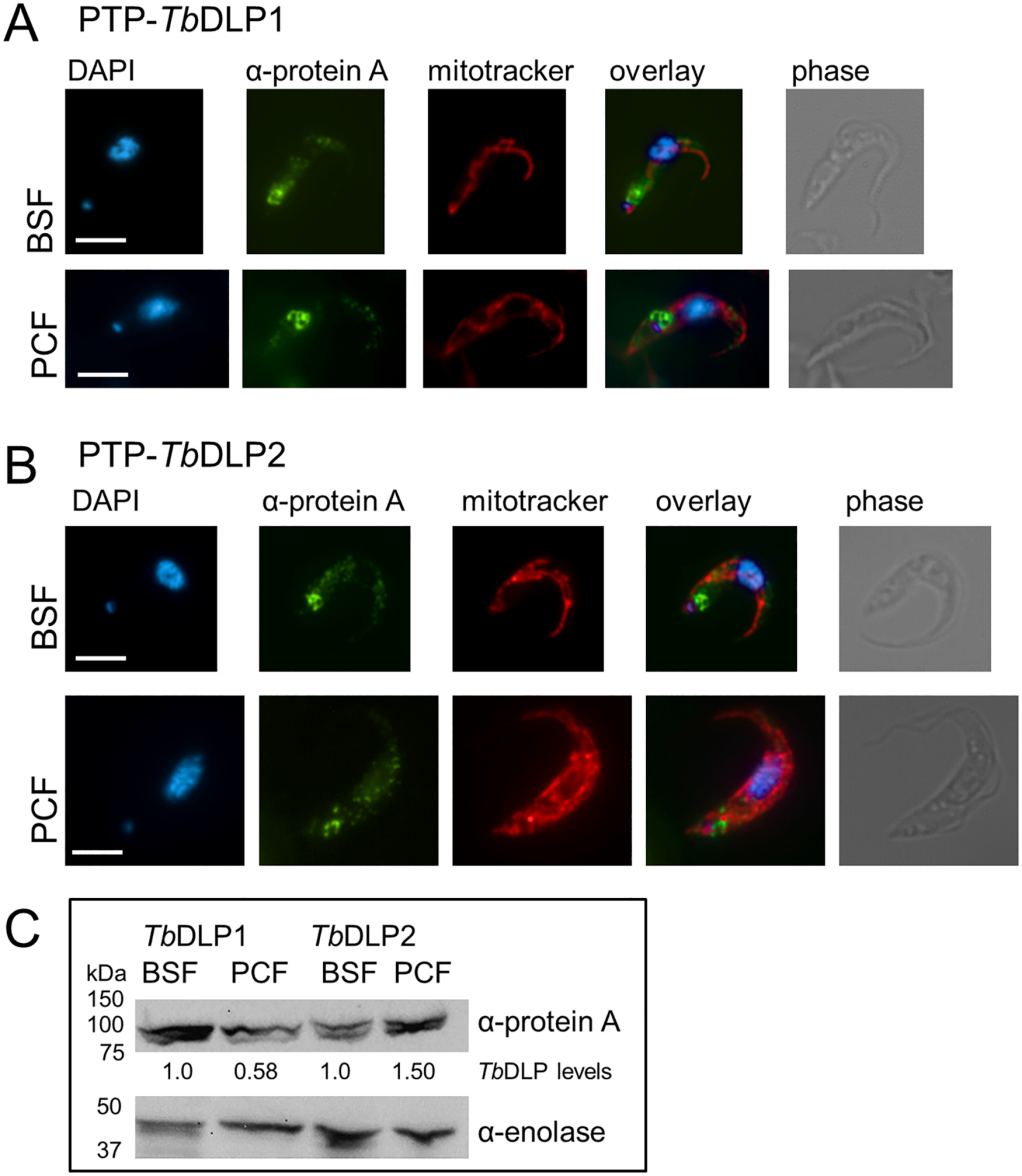
Localization and differential abundance of *Tb*DLP1 and *Tb*DLP2. A) Localization of *Tb*DLP1in BSF and PCF. Immunofluorescence analysis of endogenously PTP-tagged *Tb*DLP1. DAPI, mitotracker (red) and anti-protein A antibody (green), an overlay of the three and a phase contrast image is shown for each cell line and life cycle stage. Scale bar = 5μm. B) Localization of *Tb*DLP2 in BSF and PCF. Immunofluorescence analysis of endogenously PTP-tagged *Tb*DLP2. DAPI, mitotracker (red) and anti-protein A antibody (green), an overlay of the three and a phase contrast image is shown for each cell line and life cycle stage. Scale bar = 5μm. C) Abundance of endogenously tagged *Tb*DLP1 and *Tb*DLP2 in BSF and PCF. Equivalent numbers of cells were loaded and PTP-tagged *Tb*DLP1 and *Tb*DLP2 protein was detected with anti-protein A antibody. Anti-enolase was used as a loading control. Band intensities were measured in ImageJ, *Tb*DLP levels in BSF were set to 1.0 and the PCF levels were calculated accordingly.

To specifically localize *Tb*DLP1 and *Tb*DLP2 in our immunofluorescence assays (Fig 2A and 2B), we introduced an N-terminal *in situ* PTP tag into the genome of both BSF and PCF cells. The N-terminus was chosen because it encompasses the invariable region of *Tb*DLP1 and *Tb*DLP2, thus precluding the accidental generation of a hybrid protein as might have occurred with either a *Tb*DLP1 or *Tb*DLP2- specific construct. This strategy also allowed easy identification of the tagged protein by PCR analysis of the genomic DNA (S1 Fig). Both *Tb*DLP1 and *Tb*DLP2 showed a punctate cytosolic distribution in either life cycle stage with a more concentrated signal at the posterior end of the cell in the flagellar pocket region (Fig 2A and 2B), which is consistent with the previous results using overexpressed *Tb*DLP-HA for localization in PCF [27]. These results, however, do not correlate with those obtained using an antibody generated against recombinant *Tb*DLP that localized the protein to the mitochondrion [24].

Making use of the available PTP-tagged *Tb*DLP bearing BSF and PCF cell lines (S1 Fig), we performed a western blot, loading equal cell numbers and showing a lower abundance of *Tb*DLP1 (about 58%) and a higher abundance of *Tb*DLP2 (about 150%, Fig 2C) in PCF. While we cannot exclude that these expression levels were influenced by the additional presence of the PTP tag, they correlate with mass spectrometry data from numerous studies [29–31, 33, 34] (Table 1). Moreover, the addition of an epitope tag was necessary to discern the two *Tb*DLP isoforms in this study.

### Characterisation of RNAi and rescue cell lines in BSF cells

To analyze the function of the *Tb*DLPs separately, we decided to express RNAi-resistant versions of each protein in the background of a *Tb*DLP-RNAi cell line. We designed our RNAi construct to target a region within the very first 512 nucleotides, since these are exactly identical between the two paralogs. We had the same nucleotides recoded to be refractory to RNAi and cloned these together with the remainder of either *Tb*DLP1 or *Tb*DLP2 into a vector designed for protein overexpression with an additional C-terminal V5-tag [40].

**Fig 3:**
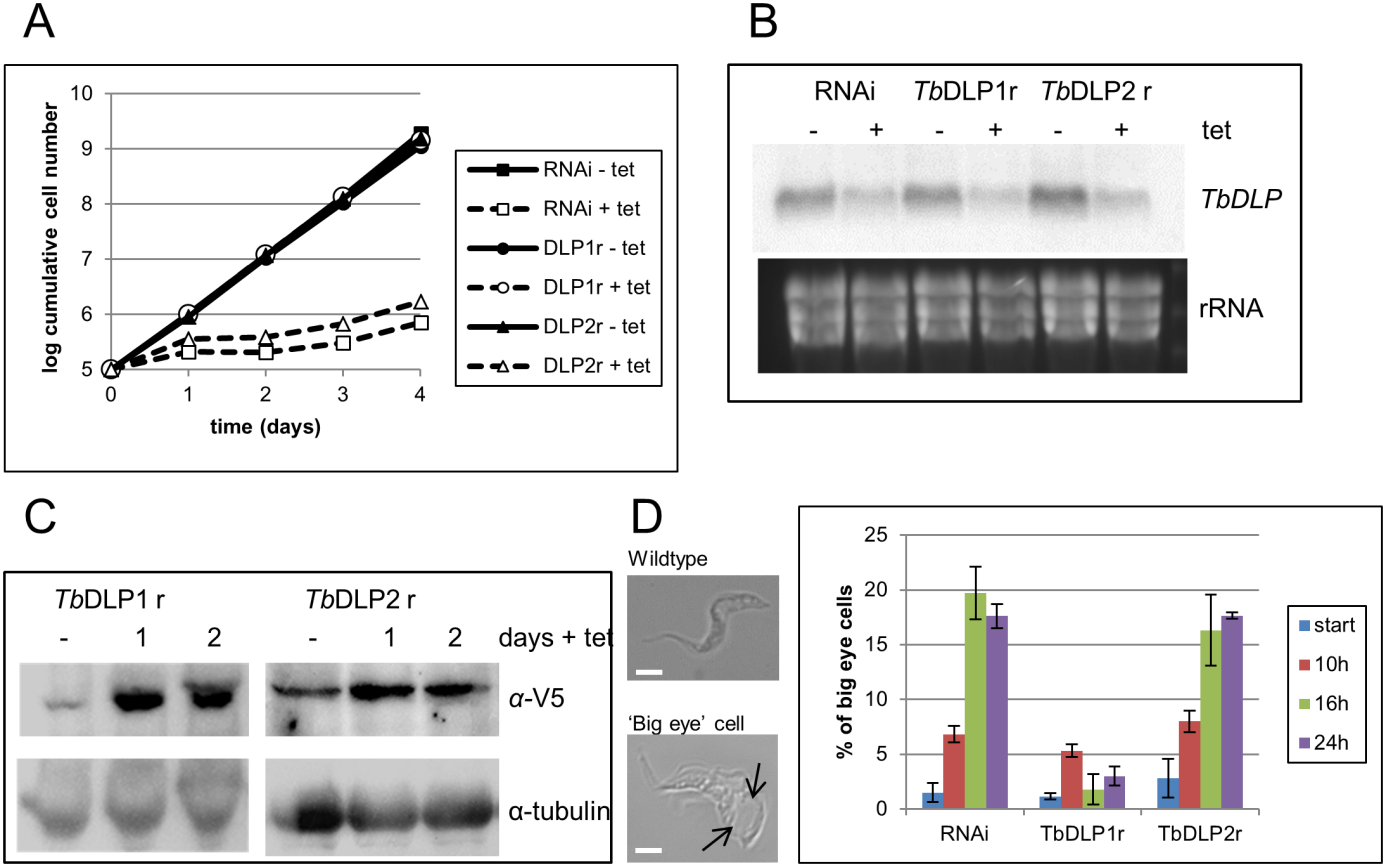
Characterization of *Tb*DLP RNAi and rescue cell lines in BSF. A) Cumulative growth of a *Tb*DLP RNAi (squares), *Tb*DLP RNAi + *Tb*DLP1 rescue (*Tb*DLP1r; dots) and *Tb*DLP RNAi + *Tb*DLP2 rescue (*Tb*DLP2r; triangles) cell line. The presence of tetracycline is indicated by open symbols and dashed lines, while the absence is indicated by solid symbols and unbroken lines. B) Northern blot of the respective cell lines shown in A). Cell lines were induced for 24 hours with tetracycline before sample preparation. The blot was probed with the *Tb*DLP RNAi fragment and the ethidium bromide stained gel is shown as a loading control. C) Western blot of *Tb*DLP1 and *Tb*DLP2 rescue cell lines. Recoded, RNAi-resistant *Tb*DLP1/2 was detected with an anti-V5 antibody. Anti-tubulin antibody was used as a loading control. D) Quantification of endocytosis defects in all three cell lines. Example images of wildtype and ‘big eye’ cells are shown, scale bar = 5μm. Arrows point at the enlarged flagellar pocket. At least 200 cells per time point and cell line were scored according to the size of their flagellar pocket by light microscopy. The experiments were performed in triplicate.

As previously mentioned for BSF cells [24], induction of RNAi against *Tb*DLP caused a rapid effect on parasite growth, with proliferation starting to decrease after 24 hours of induction and cells slowly escaping the RNAi around day 3 post induction (Fig 3A). When RNAi against *Tb*DLP was induced in a cell line that also expressed a recoded version of *Tb*DLP1, both induced and non-induced cultures grew at a comparable rate (Fig 3A), proving efficiency of the selected strategy. In contrast, expression of a recoded version of *Tb*DLP2 in the same RNAi background cell line was not able to rescue the observed growth defect of *Tb*DLP depletion (Fig 3A). Depletion of the endogenous *Tb*DLP transcript was verified by northern blot analysis and was comparable in all cell lines analyzed (Fig 3B), thus ruling out a rescue simply caused by inefficient depletion of *Tb*DLP mRNA. In addition, western blot analysis showed expression of the recoded, V5-tagged proteins in both analyzed cell lines with the expression of *Tb*DLP2 being about 2 fold lower than *Tb*DLP1 in this experiment (Fig 3C). Expression levels of *Tb*DLP2 were consistently lower than for *Tb*DLP1 in different inductions and for several clones (data not shown) and we cannot exclude that this prevented rescue of the observed phenotypes. Currently, we have no explanation for this observation since both proteins are expressed using the same parental construct, which gives similar expression levels in the PCF. Since the depletion of *Tb*DLP had previously been shown to cause endocytosis defects in the PCF flagellates [27], we also quantified the so-called ‘big eyes’ in all cell lines described above over the first 24 hours of RNAi induction. As expected, *Tb*DLP RNAi caused a rapid appearance of these cell types, reaching a plateau of about 20% after 16 hours of the addition of tetracycline (Fig 3D). Similar to the effects on growth, this phenotype was also rescued by the presence of *Tb*DLP1, where a minor initial increase of endocytosis defects after 10 hours of RNAi induction was mostly overcome by 16 and 24 hours (Fig 3D). This was not the case with the expression of *Tb*DLP2, where the amount of cells with the ‘big eye’ phenotype increased steadily over the course of induction to reach a similar percentage as the *Tb*DLP RNAi-only cell lines (Fig 3D). No cell cycle defects were observed in any of the cell lines investigated and described in this study (S2A Fig). Moreover, overexpression of either *Tb*DLP in a wildtype background did not affect growth (S3A and S3B Fig), the cell cycle (S3C Fig), or endocytosis (S3D Fig).

### Characterization of RNAi and rescue cell lines in PCF cells

**Fig 4:**
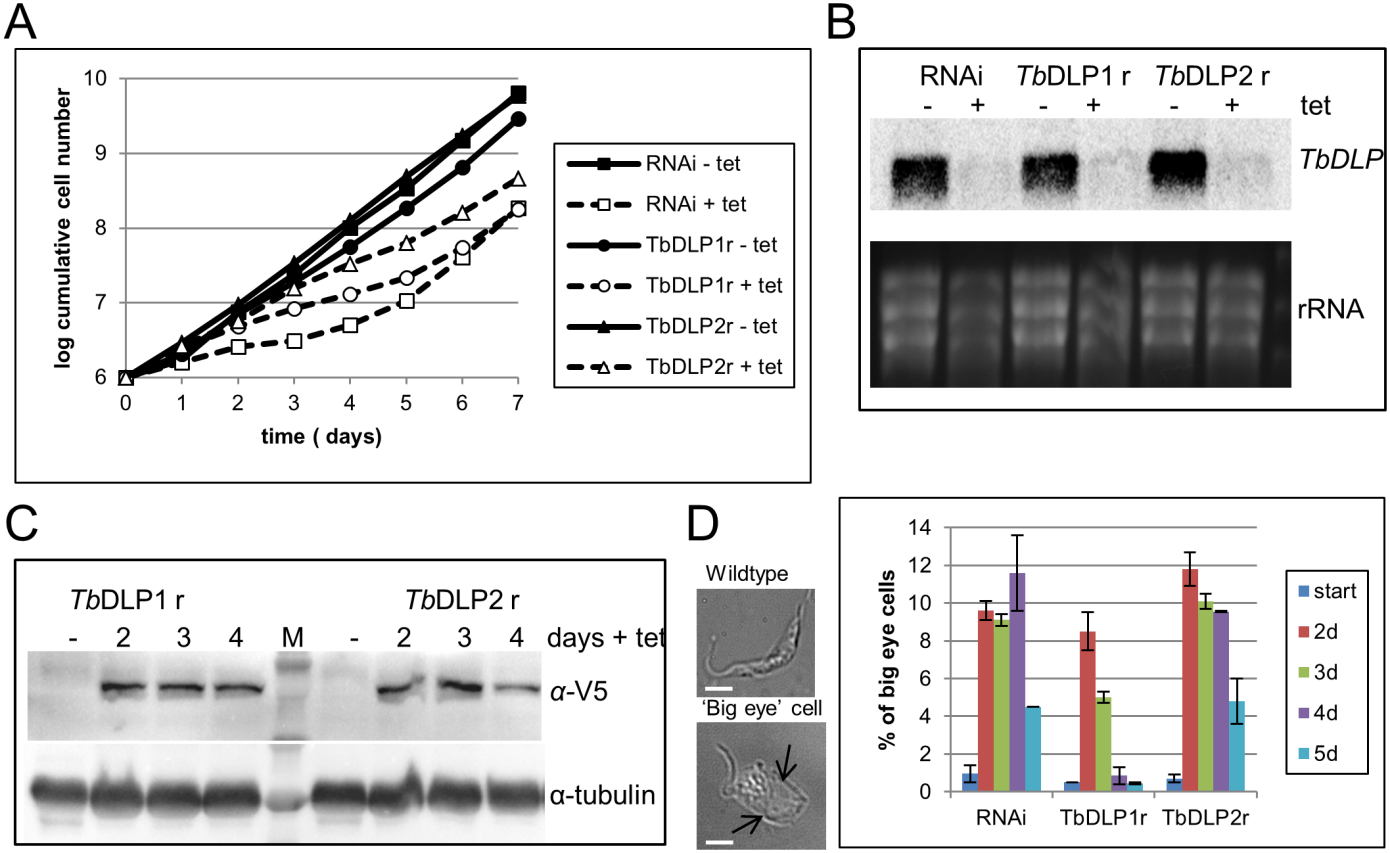
Characterization of *Tb*DLP RNAi and rescue cell lines in PCF. A) Cumulative growth of a *Tb*DLP RNAi (squares), *Tb*DLP RNAi + *Tb*DLP1 rescue (*Tb*DLP1r; dots) and *Tb*DLP RNAi + *Tb*DLP2 rescue (*Tb*DLP2; triangles) cell line. The presence of tetracycline is indicated by open symbols and dashed lines, while the absence is indicated by solid symbols and unbroken lines. B) Northern blot of the respective cell lines shown in a). Cell lines were induced for 24 hours with tetracycline before sample preparation. The blot was probed with the *Tb*DLP RNAi fragment and also with beta-tubulin as a loading control. C) Western blot of *Tb*DLP1 and *Tb*DLP2 rescue cell lines. Recoded, RNAi-resistant *Tb*DLP1/2 was detected with an anti-V5 antibody. Anti-tubulin antibody was used as a loading control. D) Quantification of endocytosis defects in all three cell lines. Example images of wildtype and ‘big eye’ cells are shown, scale bar = 5μm. Arrows point at the enlarged flagellar pocket. At least 200 cells per time point and cell line were scored according to the size of their flagellar pocket by light microscopy. The experiments were performed in triplicate.

The same *Tb*DLP RNAi and rescue experiments performed in PCF parasites gave rather different results. RNAi against the *Tb*DLP transcript caused a rapid growth defect in agreement with previous studies [24,27], but neither the expression of recoded *Tb*DLP1 nor *Tb*DLP2 could fully rescue this growth defect (Fig 4A). Instead, the growth of both induced cell lines was somewhere in-between the induced and non-induced *Tb*DLP RNAi-only cell lines, with all analyzed cell lines eventually escaping the RNAi response after day 4 post-induction. Efficiency of *Tb*DLP RNAi was monitored by northern blot and shown to be similar in all analyzed lines (Fig 4B). Expression of the V5-tagged rescue proteins was also similar and consistent over several days of induction (Fig 4C).

Performing the ‘big eye’ analysis on these cell lines at days 2, 3, 4 and 5 post RNAi-induction showed a steady increase in endocytosis defects in the *Tb*DLP RNAi-only cells until it reached about 12% at day 4 (Fig 4D). Subsequently, the percentage of visibly altered flagellates decreased again concomitant with a restart of their wild type growth (Fig 4A and 4D). *Tb*DLP1 expression did not affect the development of endocytosis defects initially until day 3; the number of big eyes then decreased a day earlier than in the *Tb*DLP RNAi-only cell line (Fig 4D). *Tb*DLP2 expression on the other hand mimicked *Tb*DLP RNAi only in this experiment (Fig 4D). None of the cell lines showed any cell cycle defects in our study (S2B Fig) in contrast to what has been reported previously [27]. We cannot exclude that this is a consequence of different RNAi vectors used (*i*.*e*. an intramolecular stem-loop RNAi construct in the study of Chanez et al. versus double T7 promoters yielding two complementary RNA strands in our study) and the resulting differences in RNAi efficiency.

**Fig 5:**
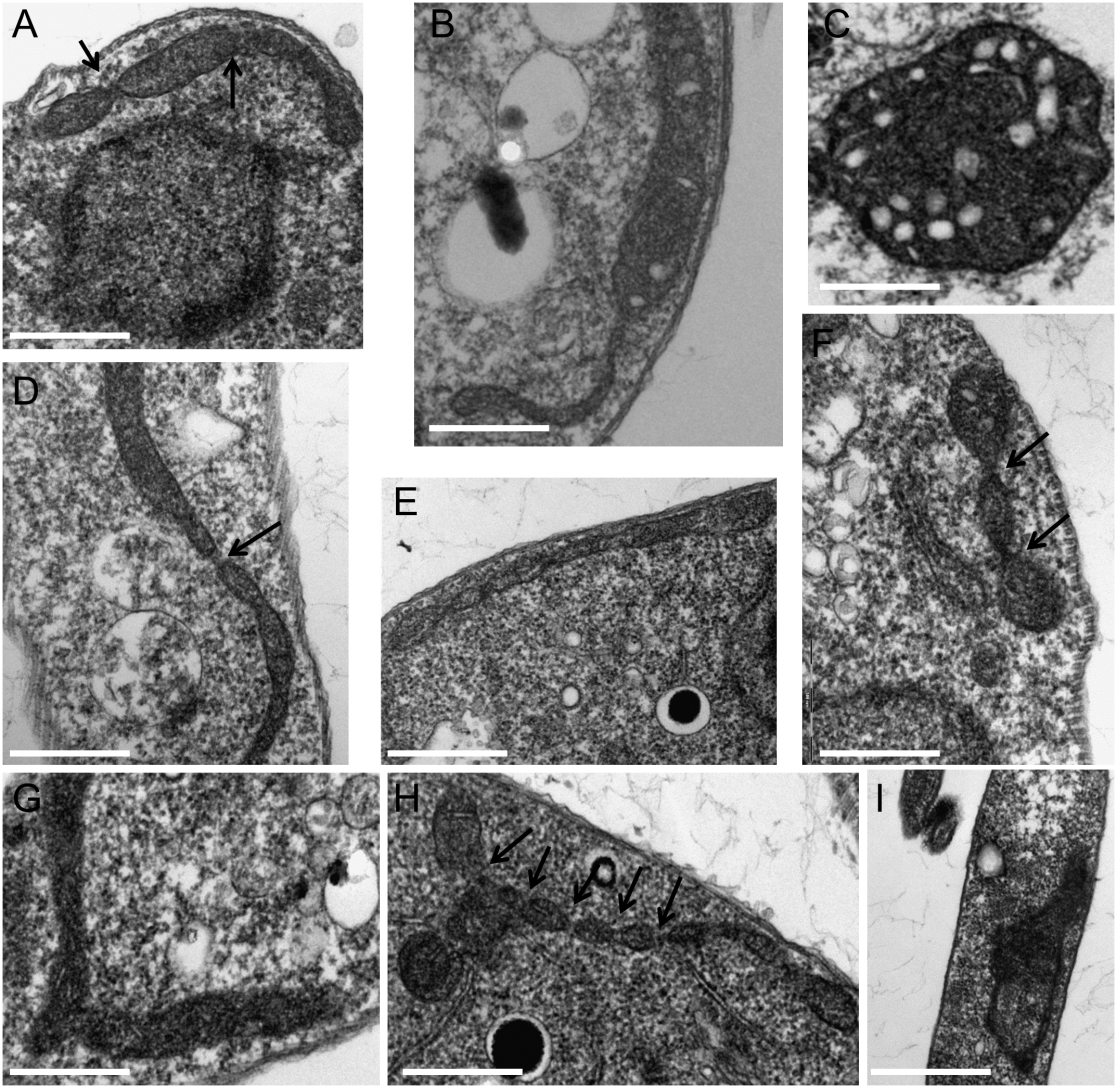
Transmission electron microscopy (TEM) analysis of PCF *Tb*DLP RNAi, *Tb*DLP1 and *Tb*DLP2 rescue cell lines. A)-C): *Tb*DLP RNAi cell line, induced for 2 (A), 3 (B) and 4 (C) days, D)-F): *Tb*DLP1 rescue cell line, induced for 2 (D), 3 (E) and 4 (F) days, G)-I): *Tb*DLP2 rescue, induced for 2 (G), 3 (H) and 4 (I) days. Scale bar = 500nm, arrows point to mitochondrial constrictions observed in all three cell lines.

To analyze any potential defects in mitochondrial division, such as an accumulation of undivided mitochondria, transmission electron microscopy (TEM) analyses were performed for all three cell lines (Fig 5). Over several days of induction of *Tb*DLP RNAi, an accumulation of vesicles within the mitochondrion (Fig 5B and 5C) in addition to visible constrictions (Fig 5; arrows) became apparent (Fig 5A–5C). The appearance of vesicles was rescued by the expression of either *Tb*DLP1 or *Tb*DLP2 as in these cell lines no such morphological alterations could be observed at any time point post RNAi-induction (Fig 5D–5I). In contrast, visible mitochondrial constrictions were still present in both rescue cell lines (Fig 5; arrows) potentially slowing down but not completely abolishing mitochondrial division, a phenomenon reported previously [24].

## Discussion

We started our investigations with the following simple hypothesis: If both highly similar *Tb*DLP proteins fulfil identical functions, why would there be any variation in sequence? Given the extremely rapid endocytosis in BSF and the much higher reliance on the process for survival in the context of antigenic variation, upregulation of a *Tb*DLP involved in scission of endocytic vesicles can be envisaged. And *vice versa*, with the PCF mitochondrion so much more metabolically active and morphologically complex than its BSF counterpart, the upregulation of machinery involved in mitochondrial division in this life stage seems appropriate. And indeed, proteomic evidence seemed to corroborate our ideas with different abundances noted for the two proteins in different life stages (Table 1 and references therein).

The family of dynamins and DLPs is huge, and members found within any one organism are usually highly specialised, at least when it comes to higher eukaryotes. Organ- and tissue-specific expression of the three mammalian dynamins has been observed, with dynamin 1 mostly found in neuronal cells, dynamin 2 being ubiquitously expressed and dynamin 3 present in the brain and testes in addition to some other tissues [50]. Differential splicing of these three dynamin isoforms generates an even bigger diversity of the expressed proteins and can also result in differential localisation within the cell [50].

The ancestral situation as is predicted for LECA, postulating one bifunctional protein for both mitochondrial and vesicle scission is supposedly also found in *T*. *brucei* and other excavate protists, as well as in some species or strains of red algae and stramenopiles [28]. However, at this point we cannot rule out that a heterodimer of the two *Tb*DLPs is responsible for both endocytosis and mitochondrial division, at least in the PCF stage. This notion is consistent with our finding that the two paralogs exhibit an apparently identical localization throughout the cell, both concentrating at the posterior end of the cell in the region of the flagellar pocket and in punctate cytosolic foci. However, our results in which RNAi-refractory *Tb*DLP1 was expressed upon simultaneous silencing of both paralogs indicate that this paralog is indeed vital and sufficient for proper endocytosis in BSF cells, regardless of its oligomeric state.

Interestingly, *Tb*DLP1 was found to be phosphorylated on two serine residues within a motif that differs between the two *Tb*DLPs [32, 33], and both studies found this post-translational modification in BSF cells. Specific interactions with potential adaptors or the activity of the DLP-containing machinery could depend on post-translational modifications present or absent from the *Tb*DLP oligomer in question, as has been reported for phosphorylation of the mitochondrial scission protein Drp1 and the endocytic activity of dynamin 1 in human neurons [51, 52]. Since different DLP adaptor proteins are used according to specific circumstances or environmental conditions in mammalian cells [53, 54], maybe a homo- or heterodimer of *Tb*DLP could also function differently in long slender BSF versus PCF, depending on specific post-translational modifications occurring at one of these *T*. *brucei* life cycle stages. Our RNAi and rescue experiments in PCF strongly suggest that neither *Tb*DLP alone can fulfil all functions essential for wild type cell growth, which is slowed down in both rescue cell lines and severely inhibited in the *Tb*DLP RNAi line. The main morphological defects observed upon depletion of *Tb*DLP, namely an increase in mitochondrial constrictions and endocytosis defects, still persist to some extent in both rescue cell lines. If we assume that a *Tb*DLP heterodimer is important for all DLP functions in PCF, the absence or depletion of one *Tb*DLP would force the remaining protein to form mostly homodimers. These might only partially fulfil the essential functions of *Tb*DLP in the cell and thus compromise growth, endocytosis and mitochondrial fission. Since endocytosis is not such an important process in PCF, the parasite might be able to handle a certain amount of downregulation without any detrimental effects on cellular functions. In contrast, the BSF flagellates generally rely on extremely rapid endocytosis and hence even minor disturbances can lead to rapid cell death. A rounding up of the BSF *Tb*DLP RNAi cells has been observed following longer induction times (data not shown), a general phenotype arising from systemic distress to the cell upon interference of essential pathways. Thus, we cannot exclude that this result could potentially mask additional, long-term effects to the mitochondrion that are specific to *Tb*DLP-depletion.

Recently, another protein homologous to the fusion GTPase mitofusin, called *Tb*MFNL (Tb927.7.2410), has been identified and characterised in *T*. *brucei* [55]. Depletion of this protein by RNAi caused a mitochondrial fenestration phenotype in BSF, suggesting an involvement in division and/or shaping the morphology of the organelle in this life cycle stage. However, depletion of *Tb*MFNL did not cause cell death, suggesting that BSF cells somehow manage to generate viable progeny presumably containing parts of the mitochondrion. Logistically, division of the BSF organelle should be a lot less challenging than that of its PCF counterpart, for which extensive branching is characteristic. It is plausible that the thin connections observed in the fenestration phenotype can be mechanically pulled apart by the ingressing cleavage furrow of the dividing cell [56]. If depletion of *Tb*MFNL is sufficient to cause mitochondrial fenestration, *Tb*DLP might play only a minor role in mitochondrial fission in BSF. Moreover, it would be interesting to analyse the phenotype of *Tb*MFNL depletion in PCF, the stage in which the protein is upregulated as compared to long slender BSF [30], and its potential interplay with *Tb*DLP in mitochondrial maintenance and inheritance.

## Supporting information

S1 Fig Verification of PTP-tagged cell lines. A) PCR analysis to verify identity of tagged *Tb*DLP in BSF. Primers binding specifically within the 3’UTR of *Tb*DLP1 (CB31) and *Tb*DLP2 (CB32) were used in combination with a primer annealing to the PTP tag (CB30). 427: wildtype cell line used as a negative control, c1, c2, c4, c5, and c6: clones obtained that expressed a PTP-tagged protein of the right size on a western blot (not shown). B) PCR analysis to verify identity of tagged *Tb*DLP in PCF. Primers binding specifically within the 3’UTR of *Tb*DLP1 (CB31) and *Tb*DLP2 (CB32) were used in combination with a primer annealing to the PTP tag (CB30). 427: wildtype cell line used as a control, B2: BSF clone 2 used as a positive control for *Tb*DLP1, B1: BSF clone 1 used as a positive control for *Tb*DLP2, c1, c3 and c4: clones obtained (not shown).
(TIF)

S2 Fig Cell cycle analysis of *Tb*DLP RNAi, *Tb*DLP1 and *Tb*DLP2 rescue cell lines. A) Cell cycle analysis for all BSF cell lines. DAPI-stained slides were scored according to the number of nuclei (N) and kinetoplasts (K). At least 200 cells per time point and cell line were analysed in triplicate. B) Cell cycle analysis for all PCF cell lines. DAPI-stained slides were scored according to the number of nuclei (N) and kinetoplasts (K). At least 200 cells per time point and cell line were analysed in triplicate.
(TIF)

S3 Fig Overexpression of *Tb*DLP1 and *Tb*DLP2 in BSF. A) Cumulative growth of a *Tb*DLP1-V5 (squares) and a *Tb*DLP2-V5 rescue (dots) cell line. The presence of tetracycline is indicated by open symbols and dashed lines, while the absence is indicated by solid symbols and unbroken lines. B) Western blot of *Tb*DLP1-V5 and *Tb*DLP2-V5 expressing cell lines. Overexpressed *Tb*DLP was detected with an antibody against the V5 tag. Anti-enolase antibody was used as a loading control. C) Cell cycle analysis for both cell lines. DAPI-stained slides were scored according to the number of nuclei (N) and kinetoplasts (K). At least 200 cells per time point and cell line were analysed in triplicate. D) Quantification of endocytosis defects in both cell lines. At least 200 cells per time point and cell line were scored according to the size of their flagellar pocket by light microscopy. The experiments were performed in triplicate.
(TIF)

S1 Table Oligonucleotides used and plasmids generated in this study. (DOCX)

S2 Table Accession numbers of kinetoplastid DLP proteins. (DOCX)

## References

[1] Matthews KR. The developmental cell biology of *Trypanosoma brucei*. J Cell Sci. 2005;118: 283–90. 10.1242/jcs.01649 15654017 PMC2686837

[2] Tielens AGM, Van Hellemond JJ. Differences in energy metabolism between Trypanosomatidae. Trends Parasitol. 1998;14: 265–271. 10.1016/s0169-4758(98)01263-0 17040781

[3] Bienen EJ, Saric M, Pollakis G, Grady RW, Clarkson ABJ. Mitochondrial development in *Trypanosoma brucei brucei* transitional bloodstream forms. Mol Biochem Parasitol.; 1991;45: 185–192. 1645458 10.1016/0166-6851(91)90085-k

[4] Tielens AGM, van Hellemond JJ. Surprising variety in energy metabolism within Trypanosomatidae. Trends Parasitol. 2009;25: 482–90. 10.1016/j.pt.2009.07.007 19748317

[5] Verner Z, Basu S, Benz C, Dixit S, Dobáková E, Faktorová D, et al Malleable mitochondrion of *Trypanosoma brucei*. Int Rev Cell Mol Biol. 2015;315: 73–151. 10.1016/bs.ircmb.2014.11.001 25708462

[6] Cross GAM, Kim HS, Wickstead B. Capturing the variant surface glycoprotein repertoire (the VSGnome) of *Trypanosoma brucei* Lister 427. Mol Biochem Parasitol.; 2014;195: 59–73. 10.1016/j.molbiopara.2014.06.004 24992042

[7] Morrison LJ, McCulloch R, Hall JPJ. DNA Recombination strategies during antigenic variation in the African trypanosome. Microbiology Spectrum. 2014; 409–435. 10.1128/microbiolspec.MDNA3-0016-2014 26104717

[8] Natesan SKA, Peacock L, Matthews K, Gibson W, Field MC. Activation of endocytosis as an adaptation to the mammalian host by trypanosomes. Eukaryot Cell. 2007;6: 2029–2037. 10.1128/EC.00213-07 17905918 PMC2168407

[9] Engstler M, Pfohl T, Herminghaus S, Boshart M, Wiegertjes G, Heddergott N, et al Hydrodynamic flow-mediated protein sorting on the cell surface of trypanosomes. Cell. 2007;131: 505–515. 10.1016/j.cell.2007.08.046 17981118

[10] Allen CL, Goulding D, Field MC. Clathrin-mediated endocytosis is essential in *Trypanosoma brucei*. EMBO J. 2003;22: 4991–5002. 10.1093/emboj/cdg481 14517238 PMC204465

[11] García-Salcedo JA, Pérez-Morga D, Gijón P, Dilbeck V, Pays E, Nolan DP. A differential role for actin during the life cycle of *Trypanosoma brucei*. EMBO J. 2004;23: 780–789. 10.1038/sj.emboj.7600094 14963487 PMC381002

[12] Field MC, Carrington M. The trypanosome flagellar pocket. Nat Rev Micro.; 2009;7: 775–786. Available: 10.1038/nrmicro2221 19806154

[13] Williams M, Kim K. From membranes to organelles: Emerging roles for dynamin-like proteins in diverse cellular processes. Eur J Cell Biol.; 2014;93: 267–277. 10.1016/j.ejcb.2014.05.002 24954468

[14] Bui HT, Shaw JM. Dynamin assembly strategies and adaptor proteins in mitochondrial fission. Curr Biol.; 2013;23: R891–9. 10.1016/j.cub.2013.08.040 24112988 PMC3832257

[15] Figueroa-Romero C, Iñiguez-Lluhí JA, Stadler J, Chang C-R, Arnoult D, Keller PJ, et al SUMOylation of the mitochondrial fission protein Drp1 occurs at multiple nonconsensus sites within the B domain and is linked to its activity cycle. FASEB J. 2009;23: 3917–27. 10.1096/fj.09-136630 19638400 PMC2775011

[16] Macdonald PJ, Francy CA, Stepanyants N, Lehman L, Baglio A, Mears JA, et al Distinct splice variants of dynamin-related protein 1 differentially utilize mitochondrial fission factor as an effector of cooperative GTPase activity. J Biol Chem. 2016;291: 493–507. 10.1074/jbc.M115.680181 26578513 PMC4697187

[17] Koirala S, Guo Q, Kalia R, Bui HT, Eckert DM, Frost A, et al Interchangeable adaptors regulate mitochondrial dynamin assembly for membrane scission. Proc Natl Acad Sci U S A. 2013;110: E1342–51. 10.1073/pnas.1300855110 23530241 PMC3625255

[18] Losón OC, Song Z, Chen H, Chan DC. Fis1, Mff, MiD49, and MiD51 mediate Drp1 recruitment in mitochondrial fission. Mol Biol Cell. 2013;24: 659–667. 10.1091/mbc.E12-10-0721 23283981 PMC3583668

[19] Guo Q, Koirala S, Perkins EM, McCaffery JM, Shaw JM. The mitochondrial fission adaptors Caf4 and Mdv1 are not functionally equivalent. PLoS One. 2012;7. 10.1371/journal.pone.0053523 PMC3534038 23300936

[20] Wang B, Nguyen M, Chang NC, Shore GC. Fis1, Bap31 and the kiss of death between mitochondria and endoplasmic reticulum. EMBO J. 2011;30: 451–452. 10.1038/emboj.2010.352 21285974 PMC3034021

[21] Bramkamp M. Structure and function of bacterial dynamin-like proteins. Biol Chem. 2012;393: 1203–1214. 10.1515/hsz-2012-0185 23109540

[22] Bürmann F, Ebert N, Van Baarle S, Bramkamp M. A bacterial dynamin-like protein mediating nucleotide-independent membrane fusion. Mol Microbiol. 2011;79: 1294–1304. 10.1111/j.1365-2958.2011.07523.x 21205012

[23] Detmer SA, Chan DC. Complementation between mouse Mfn1 and Mfn2 protects mitochondrial fusion defects caused by CMT2A disease mutations. J Cell Biol. 2007;176: 405–414. 10.1083/jcb.200611080 17296794 PMC2063976

[24] Morgan GW, Goulding D, Field MC. The single dynamin-like protein of *Trypanosoma brucei* regulates mitochondrial division and is not required for endocytosis. J Biol Chem. 2004;279: 10692–701. 10.1074/jbc.M312178200 14670954

[25] Hammarton TC. Cell cycle regulation in *Trypanosoma brucei*. Mol Biochem Parasitol. 2007;153: 1–8. 10.1016/j.molbiopara.2007.01.017 17335918 PMC1914216

[26] Wang J, Englund PT, Jensen RE. TbPIF8, a *Trypanosoma brucei* protein related to the yeast Pif1 helicase, is essential for cell viability and mitochondrial genome maintenance. Mol Microbiol. 2012;83: 471–485. 10.1111/j.1365-2958.2011.07938.x 22220754 PMC3262056

[27] Chanez A-L, Hehl AB, Engstler M, Schneider A. Ablation of the single dynamin of *T*. *brucei* blocks mitochondrial fission and endocytosis and leads to a precise cytokinesis arrest. J Cell Sci. 2006;119: 2968–74. 10.1242/jcs.03023 16787942

[28] Purkanti R, Thattai M. Ancient dynamin segments capture early stages of host-mitochondrial integration. Proc Natl Acad Sci U S A. 2015;112: 2800–5. 10.1073/pnas.1407163112 25691734 PMC4352829

[29] Butter F, Bucerius F, Michel M, Cicova Z, Mann M, Janzen CJ. Comparative proteomics of two life cycle stages of stable isotope-labeled *Trypanosoma brucei* reveals novel components of the parasite’s host adaptation machinery. Mol Cell Proteomics. 2012;12: 172–179. 10.1074/mcp.M112.019224 23090971 PMC3536898

[30] Dejung M, Subota I, Bucerius F, Dindar G, Freiwald A, Engstler M, et al Quantitative proteomics uncovers novel factors involved in developmental differentiation of *Trypanosoma brucei*. PLoS Pathog. 2016;12: 1–20. 10.1371/journal.ppat.1005439 PMC4765897 26910529

[31] Gunasekera K, Wüthrich D, Braga-Lagache S, Heller M, Ochsenreiter T. Proteome remodelling during development from blood to insect-form *Trypanosoma brucei* quantified by SILAC and mass spectrometry. BMC Genomics. 2012;13: 556 10.1186/1471-2164-13-556 23067041 PMC3545838

[32] Nett IRE, Martin DMA, Miranda-Saavedra D, Lamont D, Barber JD, Mehlert A, et al The phosphoproteome of bloodstream form *Trypanosoma brucei*, causative agent of African sleeping sickness. Mol Cell Proteomics. 2009;8: 1527–38. 10.1074/mcp.M800556-MCP200 19346560 PMC2716717

[33] Urbaniak MD, Martin DMA, Ferguson MAJ. Global quantitative SILAC phosphoproteomics reveals differential phosphorylation is widespread between the procyclic and bloodstream form lifecycle stages of *Trypanosoma brucei*. J Proteome Res. 2013;12: 2233–2244. 10.1021/pr400086y 23485197 PMC3646404

[34] Urbaniak MD, Guther MLS, Ferguson MAJ. Comparative SILAC proteomic analysis of *Trypanosoma brucei* bloodstream and procyclic lifecycle stages. PLoS One. 2012;7: e36619 10.1371/journal.pone.0036619 22574199 PMC3344917

[35] Brun R, Schönenberger. Cultivation and in vitro cloning or procyclic culture forms of *Trypanosoma brucei* in a semi-defined medium. Acta Trop. 1979;36: 289–292. 43092

[36] Coustou V, Biran M, Breton M, Guegan F, Rivière L, Plazolles N, et al Glucose-induced remodeling of intermediary and energy metabolism in procyclic *Trypanosoma brucei*. J Biol Chem. 2008;283: 16343–16354. 10.1074/jbc.M709592200 18430732

[37] Wirtz E, Leal S, Ochatt C, Cross GA. A tightly regulated inducible expression system for conditional gene knock-outs and dominant-negative genetics in *Trypanosoma brucei*. Mol Biochem Parasitol. 1999;99: 89–101. http://www.ncbi.nlm.nih.gov/pubmed/10215027 10215027 10.1016/s0166-6851(99)00002-x

[38] Changmai P, Horáková E, Long S, Černotíková-Stříbrná E, McDonald LM, Bontempi EJ, et al Both human ferredoxins equally efficiently rescue ferredoxin deficiency in *Trypanosoma brucei*. Mol Microbiol. 2013;89: 135–51. 10.1111/mmi.12264 23675735

[39] Wickstead B, Ersfeld K, Gull K. Targeting of a tetracycline-inducible expression system to the transcriptionally silent minichromosomes of *Trypanosoma brucei*. Mol Biochem Parasitol. 125: 211–6. http://www.ncbi.nlm.nih.gov/pubmed/12467990 12467990 10.1016/s0166-6851(02)00238-4

[40] Surve S, Heestand M, Panicucci B, Schnaufer A, Parsons M. Enigmatic presence of mitochondrial complex I in *Trypanosoma brucei* bloodstream forms. Eukaryot Cell. 2012;11: 183–193. 10.1128/EC.05282-11 22158713 PMC3272898

[41] Kelly S, Reed J, Kramer S, Ellis L, Webb H, Sunter J, et al Functional genomics in *Trypanosoma brucei*: a collection of vectors for the expression of tagged proteins from endogenous and ectopic gene loci. Mol Biochem Parasitol. 2007;154: 103–9. 10.1016/j.molbiopara.2007.03.012 17512617 PMC2705915

[42] Hashimi H, McDonald L, Stříbrná E, Lukeš J. Trypanosome letm1 protein is essential for mitochondrial potassium homeostasis. J Biol Chem. 2013;288: 26914–26925. 10.1074/jbc.M113.495119 23893410 PMC3772241

[43] Schneider CA, Rasband WS, Eliceiri KW. NIH Image to ImageJ: 25 years of image analysis. Nat Methods. 2012;9: 671–5. http://www.ncbi.nlm.nih.gov/pubmed/22930834 22930834 10.1038/nmeth.2089 PMC5554542

[44] Sievers F, Wilm A, Dineen D, Gibson TJ, Karplus K, Li W, et al Fast, scalable generation of high-quality protein multiple sequence alignments using Clustal Omega. Mol Syst Biol. 2011;7: 539 10.1038/msb.2011.75 21988835 PMC3261699

[45] Katoh K, Standley DM. MAFFT multiple sequence alignment software version 7: Improvements in performance and usability. Mol Biol Evol. 2013;30: 772–780. 10.1093/molbev/mst010 23329690 PMC3603318

[46] Gouy M, Guindon S, Gascuel O. SeaView version 4: A multiplatform graphical user interface for sequence alignment and phylogenetic tree building. Mol Biol Evol. 2010;27: 221–4. 10.1093/molbev/msp259 19854763

[47] Stamatakis A. RAxML version 8: A tool for phylogenetic analysis and post-analysis of large phylogenies. Bioinformatics. 2014;30: 1312–1313. 10.1093/bioinformatics/btu033 24451623 PMC3998144

[48] Ronquist F, Teslenko M, Van Der Mark P, Ayres DL, Darling A, Höhna S, et al Mrbayes 3.2: Efficient bayesian phylogenetic inference and model choice across a large model space. Syst Biol. 2012;61: 539–542. 10.1093/sysbio/sys029 22357727 PMC3329765

[49] Marks B, Stowell MH, Vallis Y, Mills IG, Gibson a, Hopkins CR, et al GTPase activity of dynamin and resulting conformation change are essential for endocytosis. Nature. 2001;410: 231–235. 10.1038/35065645 11242086

[50] Cao H, Garcia F, Mcniven MA. Differential distribution of dynamin isoforms in mammalian cells. Mol Biol Cell. 1998;9: 2595–2609. 9725914 10.1091/mbc.9.9.2595 PMC25532

[51] Kashatus DF, Lim K-H, Brady DC, Pershing NLK, Cox AD, Counter CM. RALA and RALBP1 regulate mitochondrial fission at mitosis. Nat Cell Biol. 2011;13: 1108–15. 10.1038/ncb2310 21822277 PMC3167028

[52] Tan TC, Valova VA, Malladi CS, Graham ME, Berven LA, Jupp OJ, et al Cdk5 is essential for synaptic vesicle endocytosis. Nat Cell Biol. 2003;5: 701–710. 10.1038/ncb1020 12855954

[53] Osellame LD, Singh AP, Stroud DA, Palmer CS, Stojanovski D, Ramachandran R, et al Cooperative and independent roles of Drp1 adaptors Mff and MiD49/51 in mitochondrial fission. J Cell Sci. 2016; jcs.185165-. 10.1242/jcs.185165 PMC6919635 27076521

[54] Zhang Z, Liu L, Wu S, Xing D. Drp1, Mff, Fis1, and MiD51 are coordinated to mediate mitochondrial fission during UV irradiation-induced apoptosis. FASEB J. 2016;30: 466–476. 10.1096/fj.15-274258 26432782

[55] Vanwalleghem G, Fontaine F, Lecordier L, Tebabi P, Klewe K, Nolan DP, et al Coupling of lysosomal and mitochondrial membrane permeabilization in trypanolysis by APOL1. Nat Commun. 2015;6: 8078 10.1038/ncomms9078 26307671 PMC4560804

[56] Benz C, Clucas C, Mottram JC, Hammarton TC. Cytokinesis in bloodstream stage *Trypanosoma brucei* requires a family of katanins and spastin. PLoS One. 2012;7. 10.1371/journal.pone.0030367 PMC3261199 22279588

