# Supplementary figures and images for: Dynamin-like proteins in Trypanosoma brucei: A division of labour between two paralogs?
Source: PLoS One. 2017 May 8;12(5):e0177200. doi: 10.1371/journal.pone.0177200 (PMC5421789; doi:10.1371/journal.pone.0177200)

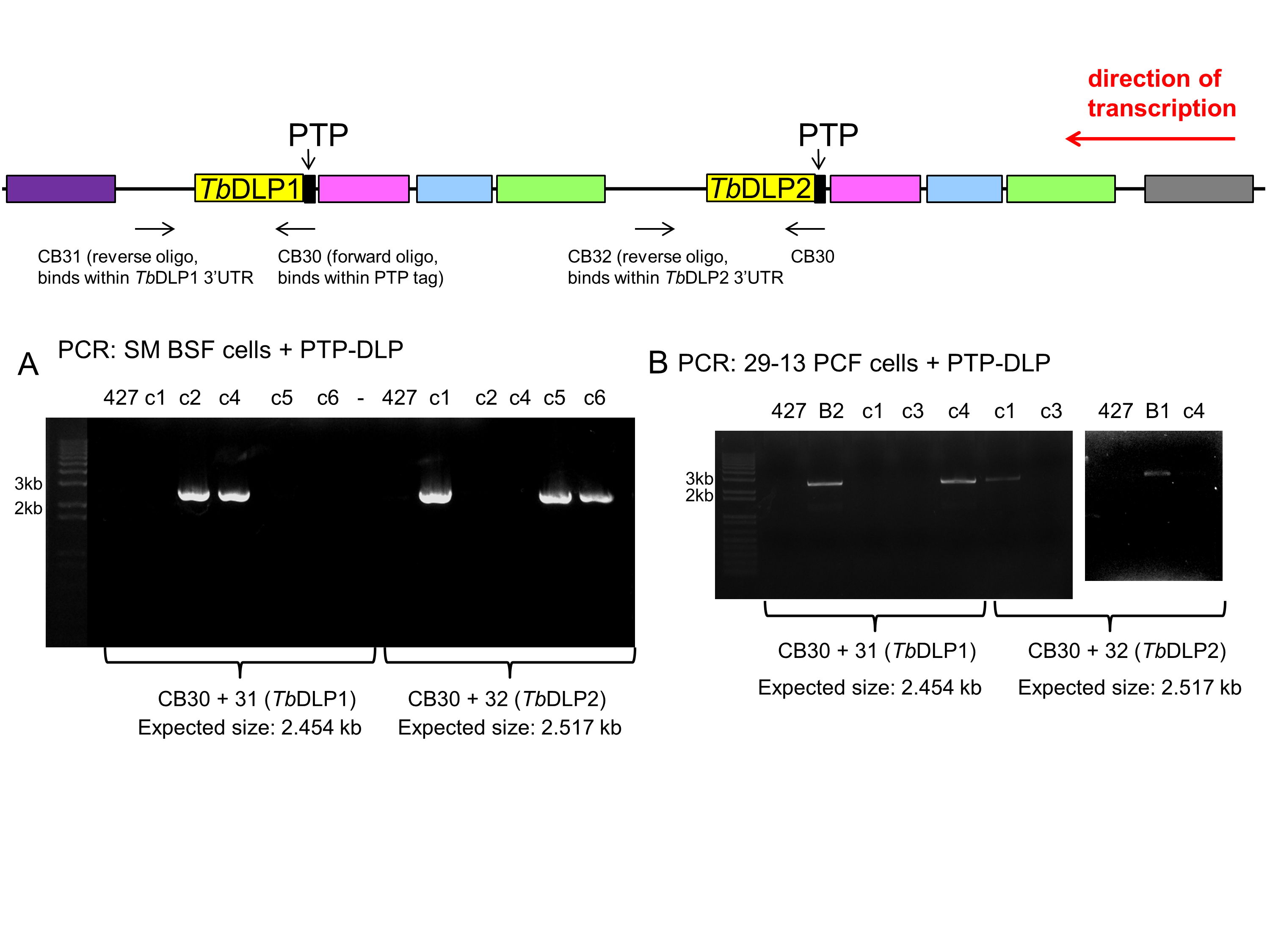

Supplement: S1 Fig — A) PCR analysis to verify identity of tagged TbDLP in BSF. Primers binding specifically within the 3’UTR of TbDLP1 (CB31) and TbDLP2 (CB32) were used in combination with a primer annealing to the PTP tag (CB30). 427: wildtype cell line used as a negative control, c1, c2, c4, c5, and c6: clones obtained that expressed a PTP-tagged protein of the right size on a western blot (not shown). B) PCR analysis to verify identity of tagged TbDLP in PCF. Primers binding specifically within the 3’UTR of TbDLP1 (CB31) and TbDLP2 (CB32) were used in combination with a primer annealing to the PTP tag (CB30). 427: wildtype cell line used as a control, B2: BSF clone 2 used as a positive control for TbDLP1, B1: BSF clone 1 used as a positive control for TbDLP2, c1, c3 and c4: clones obtained (not shown). (TIF) [file pone.0177200.s001.tif]

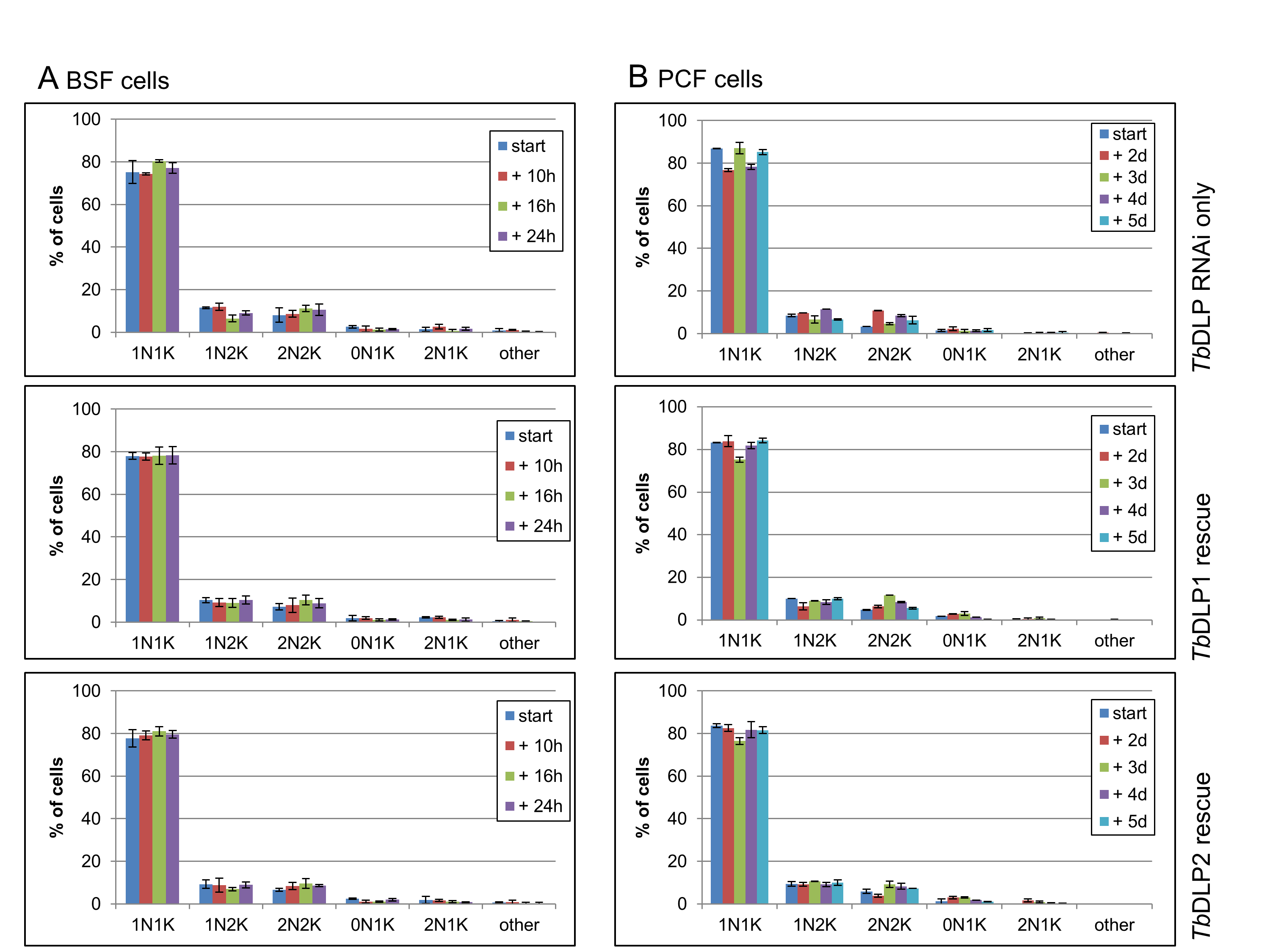

Supplement: S2 Fig — A) Cell cycle analysis for all BSF cell lines. DAPI-stained slides were scored according to the number of nuclei (N) and kinetoplasts (K). At least 200 cells per time point and cell line were analysed in triplicate. B) Cell cycle analysis for all PCF cell lines. DAPI-stained slides were scored according to the number of nuclei (N) and kinetoplasts (K). At least 200 cells per time point and cell line were analysed in triplicate. (TIF) [file pone.0177200.s002.tif]

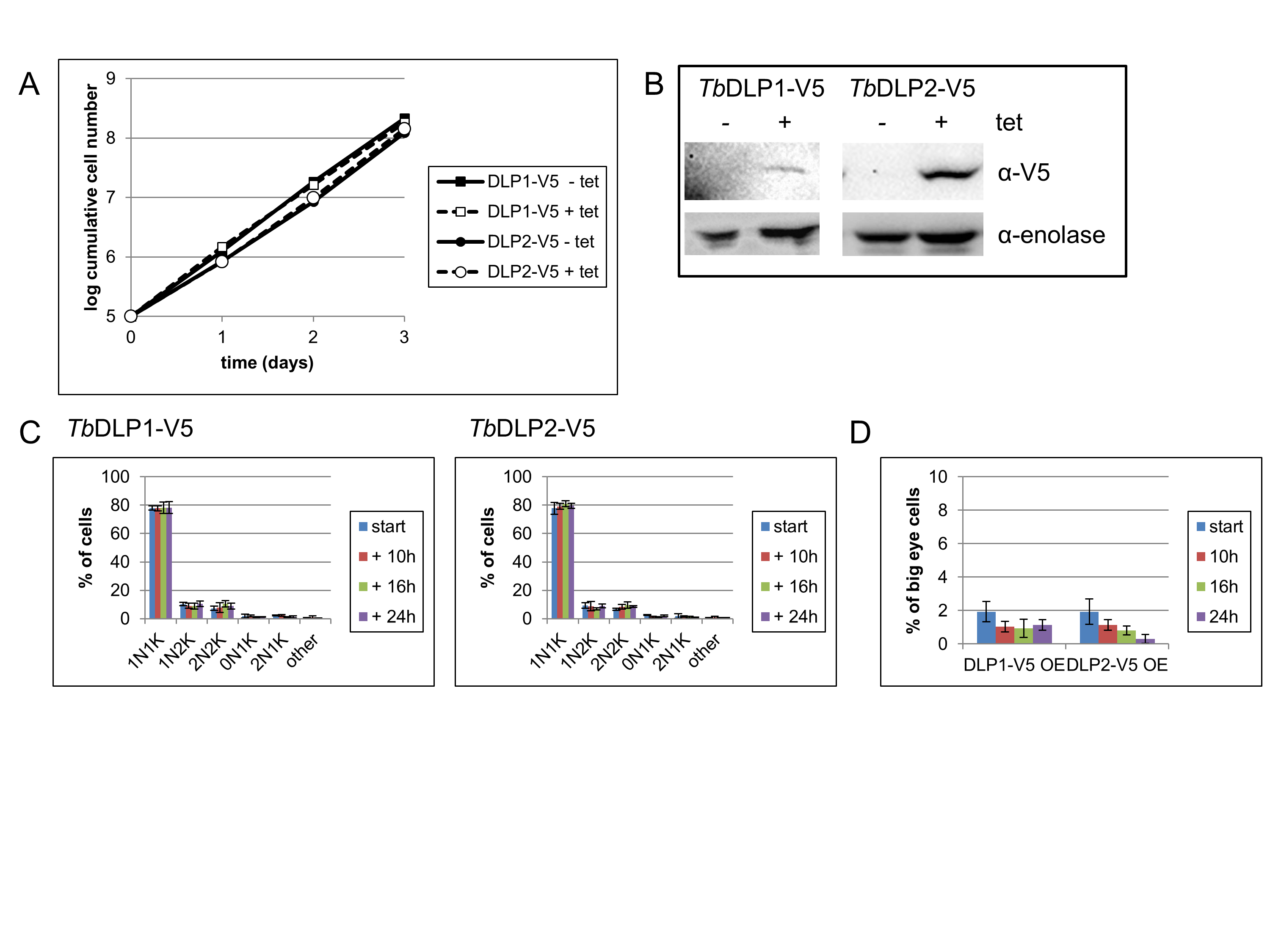

Supplement: S3 Fig — A) Cumulative growth of a TbDLP1-V5 (squares) and a TbDLP2-V5 rescue (dots) cell line. The presence of tetracycline is indicated by open symbols and dashed lines, while the absence is indicated by solid symbols and unbroken lines. B) Western blot of TbDLP1-V5 and TbDLP2-V5 expressing cell lines. Overexpressed TbDLP was detected with an antibody against the V5 tag. Anti-enolase antibody was used as a loading control. C) Cell cycle analysis for both cell lines. DAPI-stained slides were scored according to the number of nuclei (N) and kinetoplasts (K). At least 200 cells per time point and cell line were analysed in triplicate. D) Quantification of endocytosis defects in both cell lines. At least 200 cells per time point and cell line were scored according to the size of their flagellar pocket by light microscopy. The experiments were performed in triplicate. (TIF) [file pone.0177200.s003.tif]
