# Supplementary material for: Dynamin-like proteins in Trypanosoma brucei: A division of labour between two paralogs?
Source: PLoS One. 2017 May 8;12(5):e0177200. doi: 10.1371/journal.pone.0177200 (PMC5421789; doi:10.1371/journal.pone.0177200)
Supplement: S1 Table — (DOCX) [file pone.0177200.s004.docx]

**S1 Table: Oligonucleotides used and plasmids generated in this study**

| purpose | Sense oligo | Antisense oligo | Plasmid |
| --- | --- | --- | --- |
| *Tb*DLP RNAi | CB36: ctatGGATCCcctcccacagattgctgttg | CB38: atcaCTCGAGgcaagaataattgtgtttgaagg | pCR56 |
| *Tb*DLP: Endogenous tagging – PTP (N) | CB48: gatcAGATCTggagcgactcatctcggtc | CB29: tttcgttaacacggcaattgttc | pCR19 |
| *Tb*DLP: Overexpression – 3xV5 (C) | recoded bit: CB69: acgaagcttATGGAaCGtCTtATtTC  2^nd^ half: CB67: CTAgatatctcctgcaaacgctgac | recoded bit: CB70: TCAgatatcGCcAGgATgATcGTaTTaGAtG  2^nd^ half: CB68: gatAGATCTaataagagcaaactctcgaacac | pCR40 (*Tb*DLP1), pCR55 (*Tb*DLP2) |
| *Tb*DLP : Integration of PTP | CB30 (within PTP tag): GCTAAATGATGCTCAGGCGCC | CB31 (within 3’UTR of *Tb*DLP1): gactTCTAGAgagtggaaggtgaaggcaaac |  |
| *Tb*DLP: Integration of PTP | CB30 (within PTP tag): GCTAAATGATGCTCAGGCGCC | CB32 (within 3’UTR of *Tb*DLP2): gtcaTCTAGAccagaaggcatgacatttagg |  |

Oligonucleotides are given from 5’ to 3’, restriction sites are underlined.
