# Supplementary material for: Dynamin-like proteins in Trypanosoma brucei: A division of labour between two paralogs?
Source: PLoS One. 2017 May 8;12(5):e0177200. doi: 10.1371/journal.pone.0177200 (PMC5421789; doi:10.1371/journal.pone.0177200)
Supplement: S2 Table — (DOCX) [file pone.0177200.s005.docx]

**S2 Table: Accession numbers of kinetoplastid DLP proteins**

| Organism | Accession number |
| --- | --- |
| *T. b. brucei* DLP1 | Tb927.3.4720 |
| *T. b. brucei* DLP2 | Tb927.3.4760 |
| *C. fasciculata* | CFAC1_200029900 |
| *E. monterogeii* | EMOLV88_360052200 |
| *L. aethiopica* | LAEL147_000538800 |
| *L. braziliensis* | LbrM.29.2180 |
| *L. infantum* | LinJ.29.2310 |
| *L. donovani* | LdBPK_292310.1 |
| *L. major* | LmjF.29.2200 |
| *L. mexicana* | LmxM.08_29.2200 |
| *L. tarentolae* | LtaP29.2350 |
| *L. pyrrhocoris* | LpyrH10_08_2620 |
| *L. seymouri* | Lsey_0116_0100 |
| *T. b. gambiense* DLP1 | Tbg972.3.5280 |
| *T. b. gambiense* DLP2 | Tbg972.3.5320 |
| *T. congolense* | TcIL3000_0_42670 |
| *T. cruzi* | TcCLB.508153.20 |
| *T. grayi* | Tgr.37.1070 |
| *T. ranegli* | TRSC58_01376 |
| *T. vivax* | TvY486_0304114 |
| *B. saltans* | BS58690 |
